# Multi-environment evaluations reveal that peanut (*Arachis hypogaea* L.) quality traits are equally determined by genotype and environment

**DOI:** 10.3389/fpls.2026.1939285

**Published:** 2026-09-18

**Authors:** Xiao Jiang, Jianxin Zhao, Jing Xu, Dongmei Yin, Hongfeng Xie, Na Chen, Lijuan Pan, Xiangzhen Yin, Junqing Ma, Xuhong Zhao, Xiaoyuan Chi

**Affiliations:** 1 Shandong Key Laboratory of Peanut Breeding, Shandong Peanut Research Institute, Qingdao, China; 2 National Key Laboratory of Crop Genetic Improvement, Huazhong Agricultural University, Wuhan, China; 3 College of Agronomy, Henan Agricultural University, Zhengzhou, China

**Keywords:** *Arachis hypogaea* L., GGE interaction, kernel quality traits, meteorological factor, multiple environments

## Abstract

**Background:**

Peanut (*Arachis hypogaea* L.) is a widely planted cash and oil crop, and regional and meteorological factors, as the most critical environmental factors, play crucial roles in peanut kernel quality traits. To explore the relationship between peanut quality traits and environmental factors, this study dissected the quality traits and the genotype-by-environment interaction factors using seven varieties across multiple environments.

**Methods:**

Analysis of variance (ANOVA) was used to partition the variance of kernel quality traits, correlation analysis was used to examine the associations between quality traits and environmental factors, and genotype plus genotype-by-environment (GGE) biplot analysis was used to evaluate the association between genotypes and multiple environments.

**Results:**

ANOVA showed that environmental factors were the largest source of variance among the three quality traits. Correlation analysis showed that long sunshine duration, high accumulated temperature, and large mean daily temperature difference were beneficial to the accumulation of oil and protein. Oil content showed an overall trend of increasing with latitude; protein content performed best in the Huang-Huai-Hai and Yangtze River Basin production areas; and sucrose content showed an overall trend of decreasing with latitude and longitude. GGE analysis further showed that HY33 achieved optimal oil content with strong multi-environment stability, while HY21 had superior protein content and the most stable sucrose performance across multi-environments.

**Conclusions:**

This study provides theoretical guidance for adjusting planting arrangements in different regions according to different climatic conditions and peanut growing periods, which is of great significance as a guide for improving peanut quality.

## Introduction

Peanut (*Arachis hypogaea* L.) is a globally important cash and oil crop. In China, the annual sowing area of peanut exceeds 4.6 million hectares, with pod yield surpassing 17 million tons, ranking first in the world (FAOSTAT, http://faostat.fao.org). As a rich source of protein, vegetable oils, vitamins, and minerals, peanuts serve as a crucial supply of edible oil and dietary protein for humans. Although peanut cultivation is widely distributed across China, distinct environmental conditions prevail in different regions, making peanut quality highly susceptible to environmental influences. Specifically, peanut quality is closely associated with climatic resources such as temperature, precipitation, and sunshine in the cultivation area. Thus, identifying peanut varieties suitable for cultivation in specific regions remains a critical challenge, and addressing this issue is of great significance for ensuring China’s oil crop security, increasing farmers’ income, and promoting the sustainable development of the peanut industry.

Extensive studies confirm genetics govern peanut quality, yet environmental effects remain non-negligible (Dwivedi et al., 2000). The regulatory mechanisms by which meteorological factors affect crop quality traits are relatively complex. Meteorological factors (light, temperature, water) modulate crop development, strongly altering yield, agronomic performance and quality in wheat, rice and maize (Han et al., 2023; Huang et al., 2016; Vida et al., 2022; David Kulyakwave et al., 2020; Jiayu et al., 2018; Wang et al., 2025; Zhang et al., 2025). Many crops have adopted agro-climatic zoning with critical climatic indicators to assess climatic suitability for cultivation (Wei et al., 2022). Relevant zoning cases include Ningxia wine grape regionalization using effective accumulated temperature (Wang et al., 2020) and crop zoning based on ≥10 °C accumulated temperature combined with fuzzy evaluation (Zhao et al., 2019). Likewise, peanut yield and agronomic traits are jointly shaped by environment and genotype-by-environment (G×E) interaction. Long-term nationwide data proved temperature, rainfall and sunshine considerably affect peanut yield (Wang et al., 2013), with growing-season precipitation as the dominant limiting factor for spring peanut yield (Chen et al., 2019). Environmental conditions exert especially strong influences on peanut oil and protein concentrations, highlighting the necessity of precise precipitation management (Kebede and Getahun, 2017). Although recent work recognizes water and sunshine as key regulators of peanut yield and quality, few reports systematically decode meteorological and geographic impacts on peanut kernel quality, lacking supporting evidence for region-specific peanut planting across China’s major peanut production areas.

Analyzing genotype-by-environment (G×E) effects of peanut varieties across diverse ecologies is a major research hotspot. Multiple statistical approaches quantify G×E interactions, including ANOVA, PCA, linear regression, AMMI, and GGE biplot (Vlachostergios and Roupakias, 2008; Serneels and Verdonck, 2008; Hristov et al., 2010; Lingaiah et al., 2020; Yan and Rajcan, 2002). The GGE biplot stands out for visualizing genotypic, environmental and G×E effects via two-dimensional graphs to screen adaptive planting zones. Current Chinese peanut climate zoning studies are constrained by limited time-series data, narrow sampling, single indicators, and ignorance of reproductive-stage differences. Widely adopted for multi-environment crop trials covering wheat, maize, rice and other crops, GGE biplot also dissects trait correlations (Jat et al., 2018; Yan and Hunt, 2001; Setimela et al., 2007; Chintalapati et al., 2019; Katuuramu et al., 2020; Bai et al., 2018; Shukla et al., 2015; Lamichaney et al., 2021; Nagesh Kumar et al., 2021; Ansarifard et al., 2020). In peanut research, this method evaluates yield, kernel quality, stress resistance, phosphorus efficiency and biochemical profiles (Oteng-Frimpong and Dakora, 2019; Tang et al., 2025; Wang et al., 2024; Zhen et al., 2024, Zhen et al., 2023). A U.S. study tested 52 peanut germplasm and confirmed Florida-grown peanuts had maximum oil and minimum protein with inverse oil–protein correlation (Wang et al., 2024). A Chinese trial with 13 peanut materials cultivated in three environments reported superior oil and protein in Weifang peanuts and peak sucrose content in Jinzhou peanuts (Tang et al., 2025). No study has yet revealed how meteorological and geographic factors shape peanut kernel quality across multiple environments. Moreover, few works identify stably adaptable varieties suitable for widespread cultivation in China’s major peanut production areas.

Aiming to explore the relationship between peanut kernel quality traits and meteorological and geographical factors, in the present study, we conducted a multi-environment evaluation using peanut varieties’ phenotypic data on the traits of oil, protein and sucrose content in 17 locations during 2013, 2014, and 2016, crossing five major peanut-producing areas in China and spanning a geographical range from 21.09°N to 43.07°N and from 106.08°E to 124.23°E, along with data on eight meteorological and geographical factors for each environment. The findings of this study will clarify the impact of meteorological factors on peanut quality and provide a scientific basis for adjusting peanut planting arrangements in different regions according to local climatic conditions and peanut growth periods.

## Materials and Methods

### Plant materials and planting locations

Huayu 19 (HY19), Huayu 21 (HY21), Huayu 30 (HY30), Huayu 33 (HY33), Jihua 10 (JH10), Jihua 9 (JH9) and Xuhua 13 (XH13) were used as test varieties. Each plot covered an area of 6.67 m^2^, consisting of 4 rows distributed across 2 ridges. The row length within each plot was approximately 4.17 m, with a total plot width of about 1.6 m. Two seed rows were sown on each ridge at a hill spacing of 15 cm, with 2 seeds per hill and approximately 110 hills per plot. The trial was arranged in a randomized complete block design with three replications for each environment.

To evaluate variety performance and multi-environment stability of quality traits under conditions representative of local farming practices, all experimental fields were selected as flat plots with moderate fertility. Field management practices including fertilization, pest and disease control, and weeding all followed routine local agronomic protocols. All experimental locations were located in monsoon regions, where the peanut growing season coincided with the rainy season; crops relied mainly on natural rainfall, and supplemental irrigation was rarely required.

To evaluate peanut kernel quality traits across multiple environments, the tested peanut varieties were planted at 17 locations covering five major peanut-producing regions across 16 provinces in China, including Shandong, Shanxi, Liaoning, Jilin, Henan, Hebei, Jiangsu, Anhui, Fujian, Jiangxi, Guangdong, Guangxi, Guizhou, Sichuan, Hunan and Hubei (**Figure 1A**) during 2013, 2014 and 2016. A total of 51 environments were used for the evaluation of peanut quality traits. Among these locations, Zhanjiang (ZJ), Ganzhou (GZ), Quanzhou (QZ) and Hezhou (HZ) belong to the South China production area; Nanchong (NC), Shaoyang (SY), Huanggang (HG) and Hefei (HF) belong to the Yangtze River Basin production area; Zhumadian (ZMD), Kaifeng (KF), Xuzhou (XZ), Weifang (WF), Baoding (BD) and Fenyang (FY) belong to the Huang-Huai-Hai production area; Jinzhou (JZ) and Siping (SP) belong to the Northeast Plain production area; Guiyang (GY) belongs to the Yunnan-Guizhou Plateau production area (**Figure 1A**).

**Figure 1:**
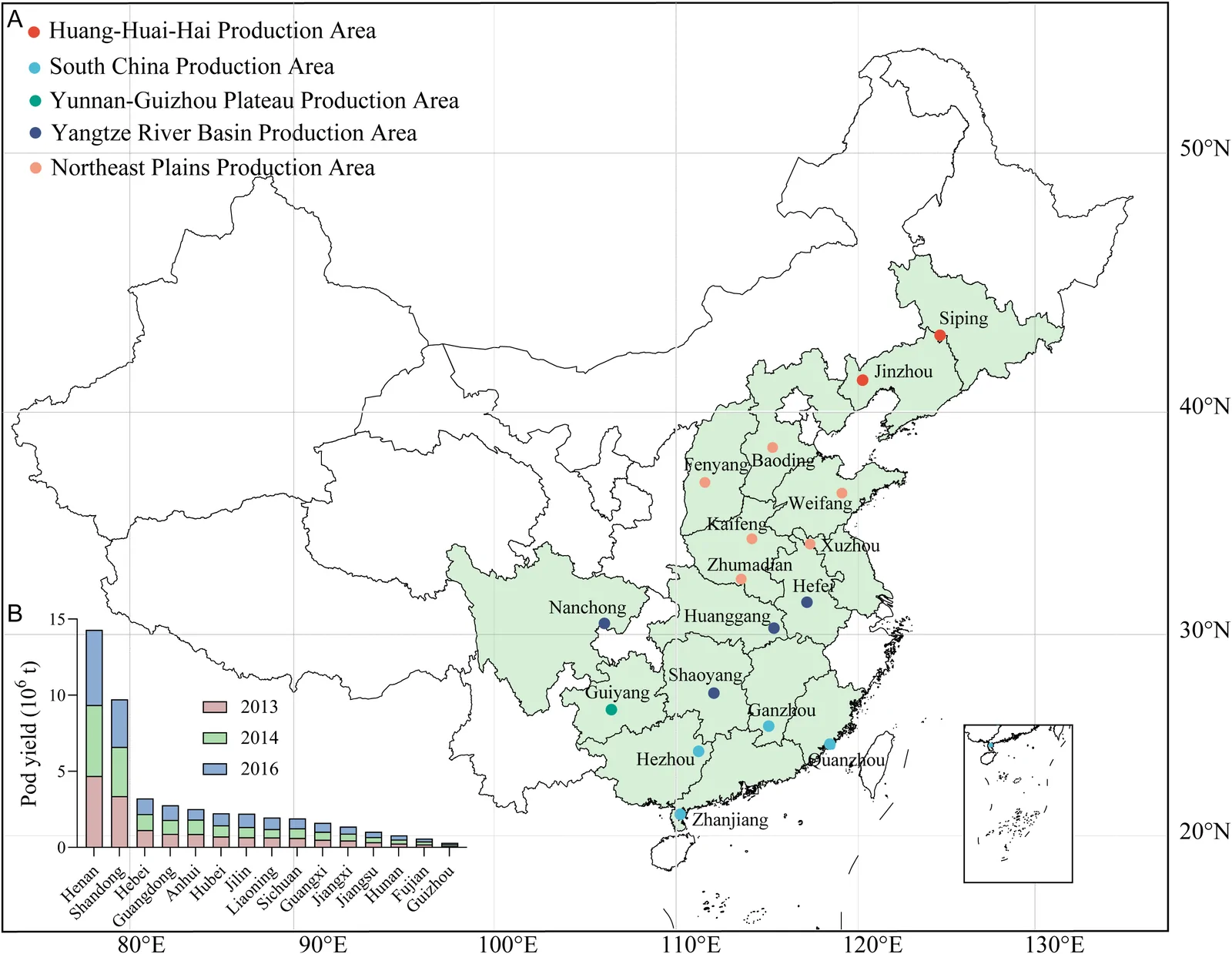
Experimental location arrangement and peanut pod yield in China. **(A)** Experimental location arrangement. **(B)** Histogram of peanut pod yields in different experimental provinces in 2013, 2014 and 2016 in China. (China National Bureau of Statistics, http://www.stats.gov.cn/).

### Multi-environment oil, protein and sucrose contents identification and statistical analysis

To accurately evaluate the peanut quality traits of the seven varieties harvested from 51 environments, oil, protein and sucrose contents were determined in accordance with China National Standard (CNS), using the Soxhlet extraction method (AOAC 995.19), Kjeldahl nitrogen determination method (AOAC Ac 4-91) and acid hydrolysis method (AOAC 920.184), respectively. Phenotypic data of the 3 peanut quality traits for each variety in every environment were collected all the three biological replicates, and statistically analyzed using Microsoft Office 2019. Meanwhile, the phenotypic data for each variety in every environment were averaged using three biological replicates for the followed analysis.

### Meteorological data collection and statistical analysis

Meteorological data for each test location in 2013, 2014 and 2016 were obtained from the China Meteorological Data Network (http://data.cma.cn). Meteorological factors across the entire peanut growth period, from sowing to harvest, in 51 environments were statistically analyzed, including accumulated temperature ≥ 10 °C, total precipitation and total sunshine duration. The mean daily temperature difference and mean daily temperature during the entire growth period for each environment were also calculated.

### Linear regression and correlation analysis between peanut quality traits and environmental factors

To elucidate the contributions of environmental and genetic factors to variations in peanut quality traits across multiple environments, two‑way ANOVA (genotype, G; environment, E) was performed for oil, protein, and sucrose contents across 51 environments using the *aov* function in RStudio (R version 4.4.2).

To investigate the relationships between quality traits and environmental factors, Pearson correlation analysis, which was implemented using the ggcorplot and corplot packages in R Studio (R version 4.4.2), was employed to analyze and visualize the correlations between peanut quality traits and environmental factors across 51 environments. Environmental factors, which consisted of geographical variables including longitude, latitude and altitude as well as meteorological variables including accumulated temperature ≥ 10 °C, total precipitation, total sunshine duration, mean daily temperature difference and mean daily temperature during the entire growth period, were used in the correlation analysis.

### GGE Biplot analysis for oil, protein and sucrose contents traits

Phenotypic data of peanut oil, protein and sucrose contents in the 51 environments were conducted GGE biplot analysis using the GGE Biplots GUI package in RStudio (R version 4.4.2). A combined analysis of variance was conducted according to the experiment design, with genotypes and locations as fixed effects. Variation in the three peanut quality traits was partitioned into parts due to environment, genotype, G×E interaction, and error. Duncan’s multiple range test (DMRT) was utilized for multiple comparison. The GGE biplot analysis was utilized for environmental vector, which-won-where, discrimination ability vs. representativeness and mean vs. stability analysis. The GGE biplot, which was constructed using the first two principal axes (Axis 1 and Axis 2), was derived from singular value decomposition (SVD) of environment-centered data that contained the GGE signal. The GGE biplot model based on SVD is expressed as follows:


$$Y_{ij}=\mu +\beta_{j}+\sum_{k=1}^{k}\lambda_{k}\gamma_{ik}\delta_{jk}$$

Where *Y_ij_* is the mean value of genotype *i* in environment *j*; *μ* is the grand mean value, *β_j_* is the main effect of environment *j*; *k* is the number of axes for adequately representing GGE variation, *n* is the singular value; λ*_k_* is the singular value of the *k*th axis (AXIS*_k_*). γ*_ik_* and *δ_jk_* are the scores of genotype *i* and environment *j* on the k-th axis, respectively.

## Results

### Multiple environments evaluation of peanut quality traits

Oil, protein, and sucrose contents were identified across 51 environments during three years. With respect to geographical factors, HG, FY, and QZ exhibited the highest oil content (**Figures 2A**, **3A**). Similarly, HF, GZ, and NC were the top three locations for protein content (**Figures 2B**, **3B**). Meanwhile, ZJ, HZ, and GY showed the highest sucrose content (**Figures 2C**, **3C**).

**Figure 2:**
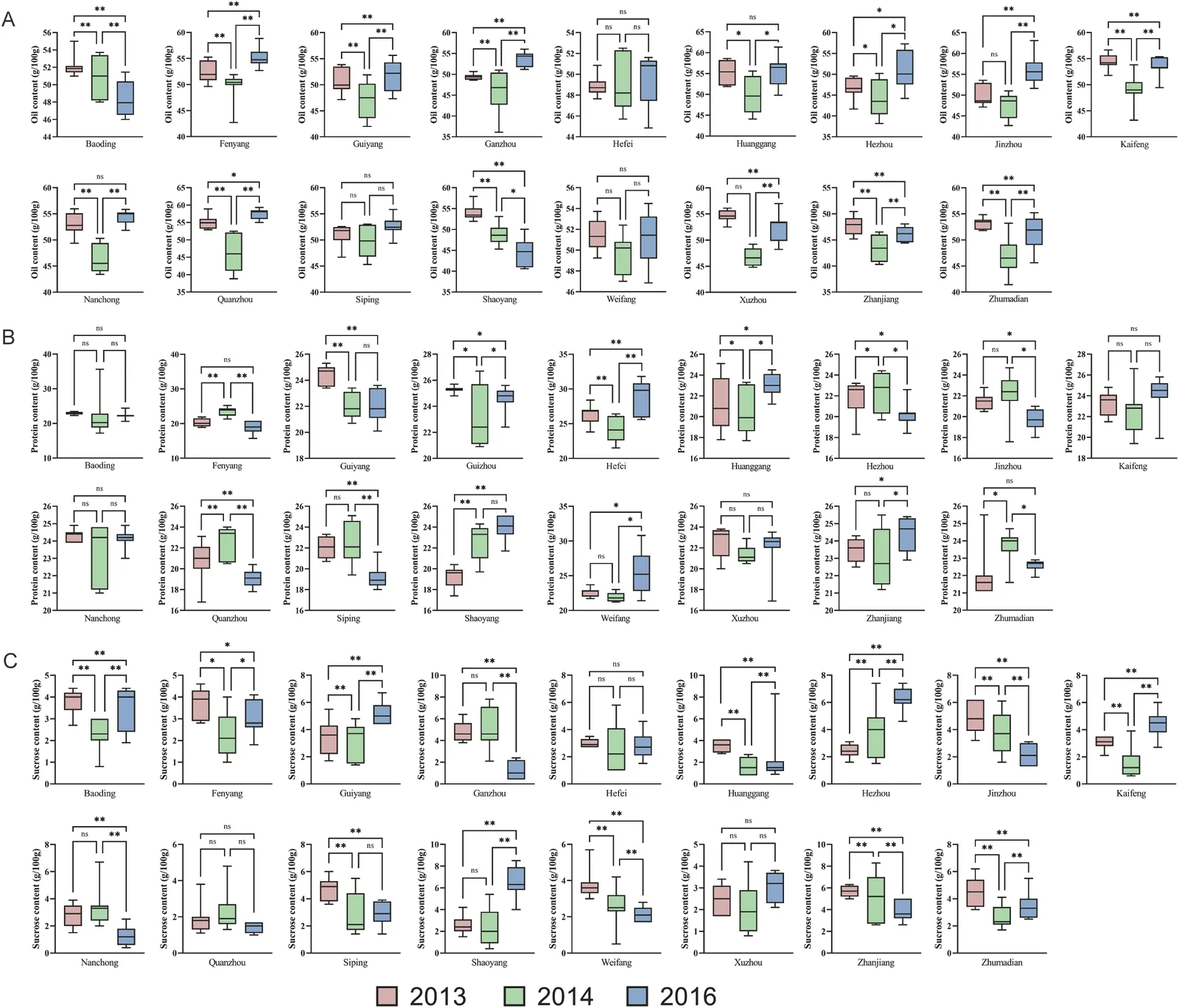
Statistical analysis of phenotypic data of peanut quality traits in multiple environments. Oil **(A)**, protein **(B)** and sucrose **(C)** content performances in 17 locations during 3 years. ns, *, and ** were not significantly, significantly at 0.05 and 0.01 level, repectively.

**Figure 3:**
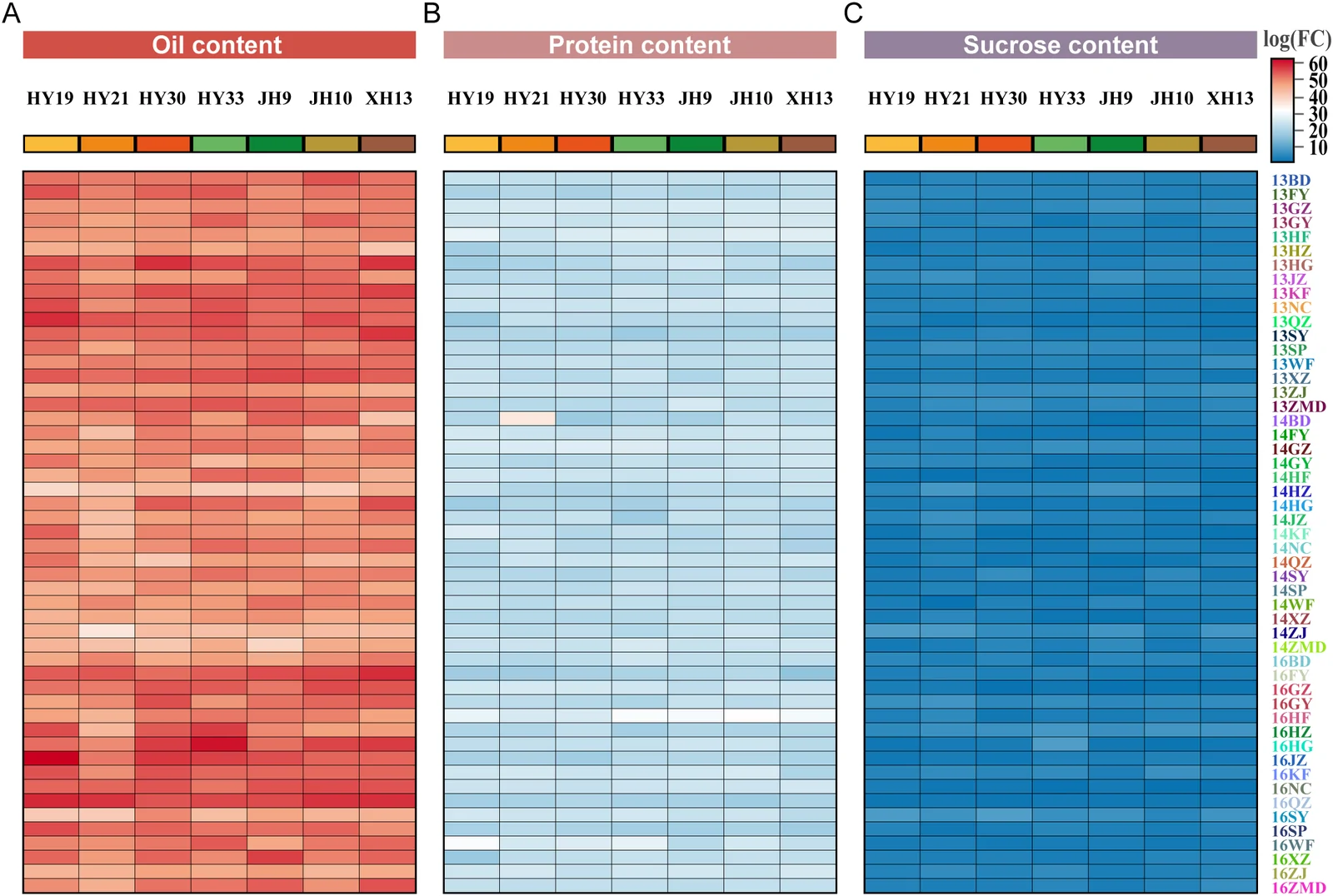
Multi-environmental phenotypic performances for quality traits of varieties. Oil **(A)**, protein **(B)** and sucrose **(C)** content of different genotypes in 51 environments.

Regarding the performance of the seven peanut varieties across 51 environments, HY33 presented the highest oil content (51.19%), with the maximum value observed at HG (**Figure 3A**). JH9 had the highest protein content (22.77%), reaching the peak at HF (**Figure 3B**). Likewise, HY21 displayed the highest sucrose content (3.71%), with the maximum value recorded at ZJ (**Figure 3C**).

### Multi-environmental meteorological data statistical analysis

Statistical analysis of meteorological data collected across three years from 17 locations revealed that NC, ZJ, HZ, TY, and SY had the maximum values of accumulated temperature ≥10 °C, precipitation, sunshine duration, mean daily temperature difference, and mean daily temperature over the entire growth period, respectively. Across all 51 environments, accumulated temperature and sunshine duration peaked in 2016, while precipitation and mean daily temperature reached their highest levels in 2013. The mean daily temperature difference remained consistent across the three experimental years (**Table 1**).

**Table 1:** Environmental factors information of each location from sowing to harvest.

Year	Location	Environment	Planting date	Harvesting date	Alt (m)	Lat	Lon	≥10°C Accumulated temperature (°C)	Sunshine duration (h)	Precipitation	Mean diurnal temperature range (°C)	Mean temperature (°C)
						(°)	(°)			(mm)		
2013	Baoding	2013BD	May. 19	Sep. 14	16.8	38.44	115.29	2947.3	579	517.7	9.85	24.77
	Fenyang	2013FY	May. 8	Sep. 24	746	37.04	111.57	3171	704.7	537.8	11.374	22.65
	Ganzhou	2013GZ	May. 29	Sep. 10	137.5	25.52	115	3060.4	745.8	354.9	8.131	29.15
	Guiyang	2013GY	Apr. 19	Sep. 9	1223.8	26.35	106.44	3076.4	642.9	603.7	8.065	21.43
	Hefei	2013HF	Jun. 15	Oct. 18	27	31.47	117.18	3211.7	753.6	559.1	7.883	27
	Hezhou	2013HZ	Apr. 26	Jul. 24	148.7	24.25	111.3	2362.2	413	671.7	7.646	26.25
	Huanggang	2013HG	Jun. 5	Aug. 29	123.8	30.44	115.4	2487.8	694.1	676.5	9.574	28.6
	Jinzhou	2013JZ	May. 17	Sep. 28	174.3	41.33	120.26	3085.3	964.8	222.5	11.788	22.85
	Kaifeng	2013KF	May. 10	Sep. 11	73.7	34.47	114.18	3359.6	709	225.7	8.774	26.88
	Nanchong	2013NC	Apr. 13	Aug. 26	347	30.45	106.08	3608	857.8	651.7	8.738	26.53
	Quanzhou	2013QZ	Aug. 10	Dec. 8	21.8	24.54	118.55	2880.1	821	238.6	4.689	23.8
	Shaoyang	2013SY	May. 4	Sep. 2	100	27.27	112.1	3449.2	703.6	361.7	9.34	28.27
	Siping	2013SP	May. 22	Sep. 26	179.5	43.07	124.23	2771	808.4	531.3	9.848	21.65
	Weifang	2013WF	May. 5	Sep. 16	22.2	36.45	119.12	3364.2	969.4	383	9.904	24.92
	Xuzhou	2013XZ	May. 10	Sep. 16	41.2	34.17	117.1	3491.6	951.9	494.4	8.499	26.86
	Zhanjiang	2013ZJ	Aug. 11	Nov. 23	53.4	21.09	110.18	2653.6	576.1	672.8	6.119	25.27
	Zhumadian	2013ZMD	May. 18	Sep. 20	106.2	32.56	113.55	3460	782	502.8	8.844	27.46
2014	Baoding	2014BD	May. 27	Sep. 12	16.8	38.44	115.29	2785.5	671.4	324.1	11.219	25.56
	Fenyang	2014FY	May. 11	Sep. 27	746	37.04	111.57	3043.9	647.8	359.4	12.074	21.74
	Ganzhou	2014GZ	Apr. 2	Aug. 1	137.5	25.52	115	3237	691.2	589.2	8.186	26.53
	Guiyang	2014GY	Apr. 21	Sep. 14	1223.8	26.35	106.44	3072.2	430.4	1165.7	7.265	20.9
	Hefei	2014HF	May. 17	Sep. 30	27	31.47	117.18	3488	489.1	589.5	7.418	25.46
	Hezhou	2014HZ	Mar. 19	Jul. 15	148.7	24.25	111.3	2843.2	334.1	1199.7	6.736	23.89
	Huanggang	2014HG	Apr. 29	Aug. 29	123.8	30.44	115.4	3086.9	633.1	723.5	8.815	25.1
	Jinzhou	2014JZ	May. 13	Sep. 25	174.3	41.33	120.26	3095.8	1000.6	3085	12.988	22.76
	Kaifeng	2014KF	May. 7	Sep. 8	73.7	34.47	114.18	3239.8	754.1	170.7	9.35	25.92
	Nanchong	2014NC	Apr. 5	Aug. 22	347	30.45	106.08	3428.8	656.1	564.7	7.436	24.49
	Quanzhou	2014QZ	Aug. 15	Dec. 10	21.8	24.54	118.55	2825.2	813.7	73.4	4.496	23.94
	Shaoyang	2014SY	May. 5	Sep. 3	100	27.27	112.1	3184.8	369.2	732.4	7.414	26.1
	Siping	2014SP	May. 17	Sep. 22	179.5	43.07	124.23	2788.9	840.4	355.4	10.743	21.62
	Weifang	2014WF	May. 15	Sep. 19	22.2	36.45	119.12	3189.8	846.2	325.5	9.956	24.92
	Xuzhou	2014XZ	May. 13	Sep. 24	41.2	34.17	117.1	3402.5	857.7	480.4	8.377	25.2
	Zhanjiang	2014ZJ	Aug. 5	Nov. 5	53.4	21.09	110.18	2497.2	658.9	476.8	6.523	26.85
	Zhumadian	2014ZMD	May. 19	Sep. 21	106.2	32.56	113.55	3061.9	504.3	510.4	9.576	24.3
2016	Baoding	2016BD	May. 18	Sep. 13	16.8	38.44	115.29	3002.6	522.4	380.3	11.163	25.23
	Fenyang	2016FY	May. 10	Sep. 26	746	37.04	111.57	3122.2	595.5	436.9	12.127	22.3
	Ganzhou	2016GZ	Mar. 31	Aug. 3	137.5	25.52	115	3319	680.3	1045.4	7.999	26.34
	Guiyang	2016GY	Apr. 15	Sep. 15	1223.8	26.35	106.44	3292	576	635	7.476	21.38
	Hefei	2016HF	Jun. 17	Oct. 13	27	31.47	117.18	3149.6	656.5	679.2	7.904	26.47
	Hezhou	2016HZ	Mar. 26	Jul. 20	148.7	24.25	111.3	2841.1	373	1092.1	7.347	24.71
	Huanggang	2016HG	May. 1	Sep. 2	123.8	30.44	115.4	3197.2	727.3	1295.2	8.922	25.58
	Jinzhou	2016JZ	May. 16	Sep. 27	174.3	41.33	120.26	3119.5	982.2	394	11.96	23.11
	Kaifeng	2016KF	May. 8	Sep. 9	73.7	34.47	114.18	3226.8	727.8	408	8.889	26.03
	Nanchong	2016NC	Apr. 7	Aug. 23	347	30.45	106.08	3403.7	805.8	542.9	8.938	24.49
	Quanzhou	2016QZ	Mar. 22	Jul. 26	21.8	24.54	118.55	2791	708.1	552.3	4.043	23.65
	Shaoyang	2016SY	Jun. 14	Sep. 25	100	27.27	112.1	2929	593.1	530.2	8.688	28.16
	Siping	2016SP	May. 15	Sep. 25	179.5	43.07	124.23	2858.5	833.2	551.3	10.441	21.33
	Weifang	2016WF	May. 7	Sep. 9	22.2	36.45	119.12	3177.2	955	370.1	5.952	25.22
	Xuzhou	2016XZ	May. 5	Sep. 10	41.2	34.17	117.1	3329.4	893.4	386.9	8.443	25.81
	Zhanjiang	2016ZJ	Feb. 29	Jun. 28	53.4	21.09	110.18	2981.6	596	529.7	5.586	24.85
	Zhumadian	2016ZMD	May. 16	Sep. 11	106.2	32.56	113.55	3045	655.5	436.5	9.945	25.59

### Multiple production areas evaluation of peanut quality traits

The analysis of quality traits in different production areas revealed that Yangtze River Basin and Huang-Huai-Hai production areas had the highest oil and protein contents, as well as the lowest sucrose content (**Figure 4**). Oil content showed an overall trend of increasing with latitude, the protein content had the lowest performance in the Northeast Plains Production area, and the sucrose content showed an overall trend of decreasing with latitude.

**Figure 4:**
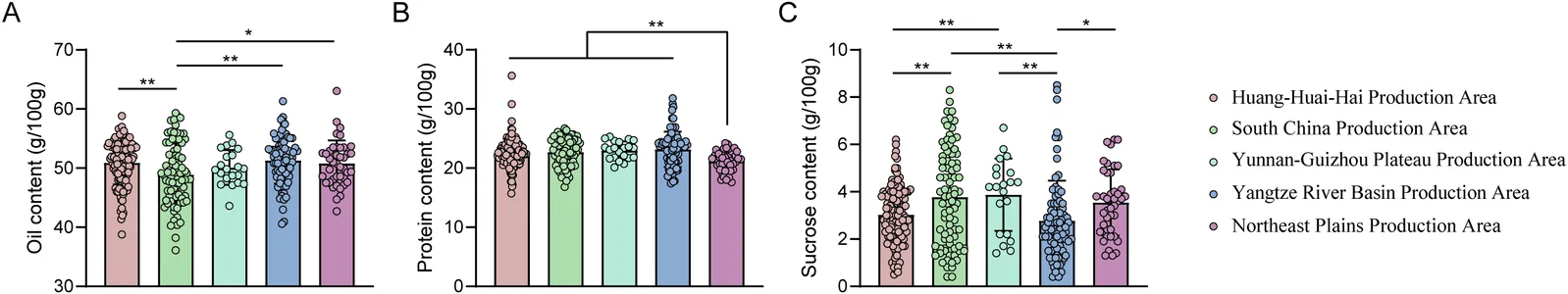
Peanut kernel quality traits performances in five production areas. Oil **(A)**, protein **(B)** and sucrose **(C)** content traits performances in five production areas.

### Multi-environmental ANOVA for genotypic and environmental factors

To elucidate the contributions of environmental and genetic factors to variations in peanut quality traits across multiple environments, two‑way ANOVA was performed for oil, protein, and sucrose contents across 51 environments. The relative contributions of G, E, and G×E to total variation were 5.22%, 68.87%, and 25.91% for oil content; 0.65%, 63.61%, and 35.74% for protein content; and 1.69%, 65.47%, and 32.84% for sucrose content, respectively (**Table 2**). The results revealed that environmental factors accounted for the largest proportion of variance for all three quality traits (all > 50%). Meanwhile, oil, protein, and sucrose contents were also substantially affected by genotype‑by‑environment interaction, indicating that environmental factors strongly influence phenotypic variation and that significant G×E interaction exists.

**Table 2:** Two-way ANOVA of genotypic and environmental factors for oil, protein and sucrose content.

Trait	Factor	Df	Sum_Sq	Mean_Sq	F_value	Pr(>F)
Oil content	G	6	330	54.96	10.06	3.91e-10***
	E	50	4357	87.14	15.95	<2e-16***
	GbyE	300	1639	5.46		
Protein content	G	6	14.3	2.379	0.907	0.49
	E	50	1400.7	28.014	10.678	<2e-16***
	GbyE	300	787.1	2.624		
Sucrose content	G	6	16.1	2.685	2.577	0.0189*
	E	50	622.9	12.459	11.959	<2e-16***
	GbyE	300	312.5	1.042		

*, ***, p at 0.05 and 0.001 level, respectively.

### Linear regression and correlation analysis between peanut quality traits and environmental factors

To examine the relationships between peanut quality traits and geographical and meteorological factors, linear regression and correlation analysis were conducted. Linear regression analysis showed that oil content was positively and linearly correlated with accumulated temperature ≥ 10 °C (R^2^ = 0.12, P = 0.011) and total sunshine duration (R^2^ = 0.13, P = 0.018) during the entire growth period. Protein content was positively and linearly correlated with mean daily temperature (R^2^ = 0.087, P = 0.031). Sucrose content was negatively and linearly correlated with latitude (R^2^ = 0.031, P = 0.097), longitude (R^2^ = 0.038, P = 0.45), accumulated temperature ≥ 10 °C (R^2^ = 0.098, P = 0.058), and total sunshine duration (R^2^ = 0.051, P = 0.12) (**Supplementary Figure S1**). The results of linear regression analysis showed that the goodness of fit between quality traits and single geographical or meteorological factor was extremely low (R^2^) and non-significant (P > 0.05), indicating that quality traits may be jointly affected by multiple factors.

Correlation analysis results showed that oil content was extremely significant positive correlated with longitude, accumulated temperature ≥ 10 °C, and total sunshine duration (p<0.01). Protein content was extremely significant positive correlated with accumulated temperature ≥ 10 °C and mean daily temperature(p<0.01), and significant positive correlated with longitude and mean daily temperature difference (p<0.01). Sucrose content was extremely significant negative correlated with longitude, latitude, accumulated temperature ≥ 10 °C, and total sunshine duration (p<0.01) (**Figure 5**).

**Figure 5:**
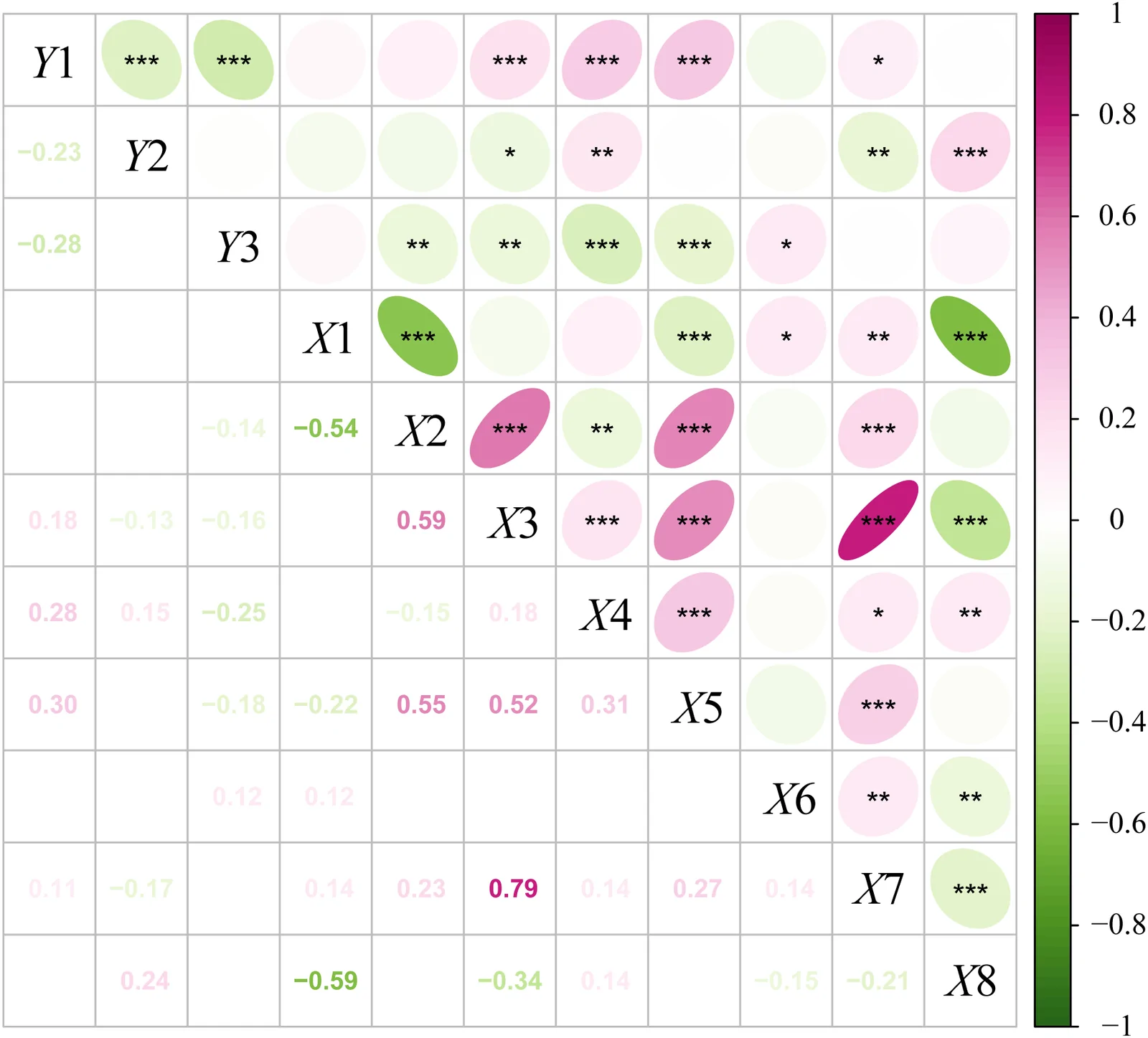
Correlation analysis between the peanut quality traits and the geographical and meteorological factors. *Y*_1_: oil content; *Y*_2_: protein content; *Y*_3_: sucrose content; *X*_1_: altitude; *X*_2_: latitude; *X*_3_: longitude; *X*_4_: the entire growth period accumulated temperature ≥10 °C; *X*_5_: the entire growth period precipitation; *X*_6_: the entire growth period sunshine duration; *X*_7_: mean daily temperature difference; *X*_8_: the entire growth period mean daily temperature. *, **, and *** were significantly at 0.05, 0.01, and 0.001 level, respectively.

### GGE Biplot analysis for peanut kernel quality traits across multi-environments

**Figure 6A** illustrates the environmental relationships for oil content. An angle< 90° between two environmental vectors indicated a positive correlation and consistent phenotypic performance, whereas an angle > 90° indicated a negative correlation. GGE biplot analysis decomposed the effects of G and G×E for quality traits across 51 environments. The first two principal axes (Axis 1 and Axis 2) explained 32.82% and 20.94% of the total phenotypic variance for oil content, respectively. Although Axis 1 and Axis 2 explained a moderate proportion of the total variance, which is typical for complex multi-environment trials, the interpretation of mega-environments should be considered with caution. KF, HG, WF, GZ, and QZ showed strong positive correlations across three years. In contrast, BD, GY, HZ, and ZMD were negatively correlated. Environments including 2016HZ, 2016JZ, 2014HG, 2016HG, and 2014KF exhibited the strongest discriminatory power for oil content among varieties. Environmental vector analysis suggested that HG and KF had stable climatic conditions and high discriminatory ability for oil content. Some locations showed similar responses, such as 2014KF and 2016HZ, as well as 2016ZMD and 2016HG, whereas 2016BD and 2016JZ were negatively correlated. The “which‑won‑where” GGE biplot grouped the 51 environments into four mega‑environments (**Figure 6B**), consisting of 22, 18, 9, and 2 environments, with XH13, HY19, HY33, and HY21 as the optimal genotypes in each mega‑environment, respectively (**Supplementary Table S2**). These results imply that the four varieties perform best in their respective mega‑environments. Environmental representativeness analysis, a key criterion for test site evaluation, revealed that 2016GY was the most representative site, followed by 2016HF, 2014FY, 2013SP) and 2014JZ (**Figure 6C**). The “Mean vs. Stability” biplot assessed overall performance and stability for oil content. HY33, XH13, and HY30 showed the highest oil content, with HY33 and HY30 exhibiting the strongest multi‑environmental stability (**Figure 6D**).

**Figure 6:**
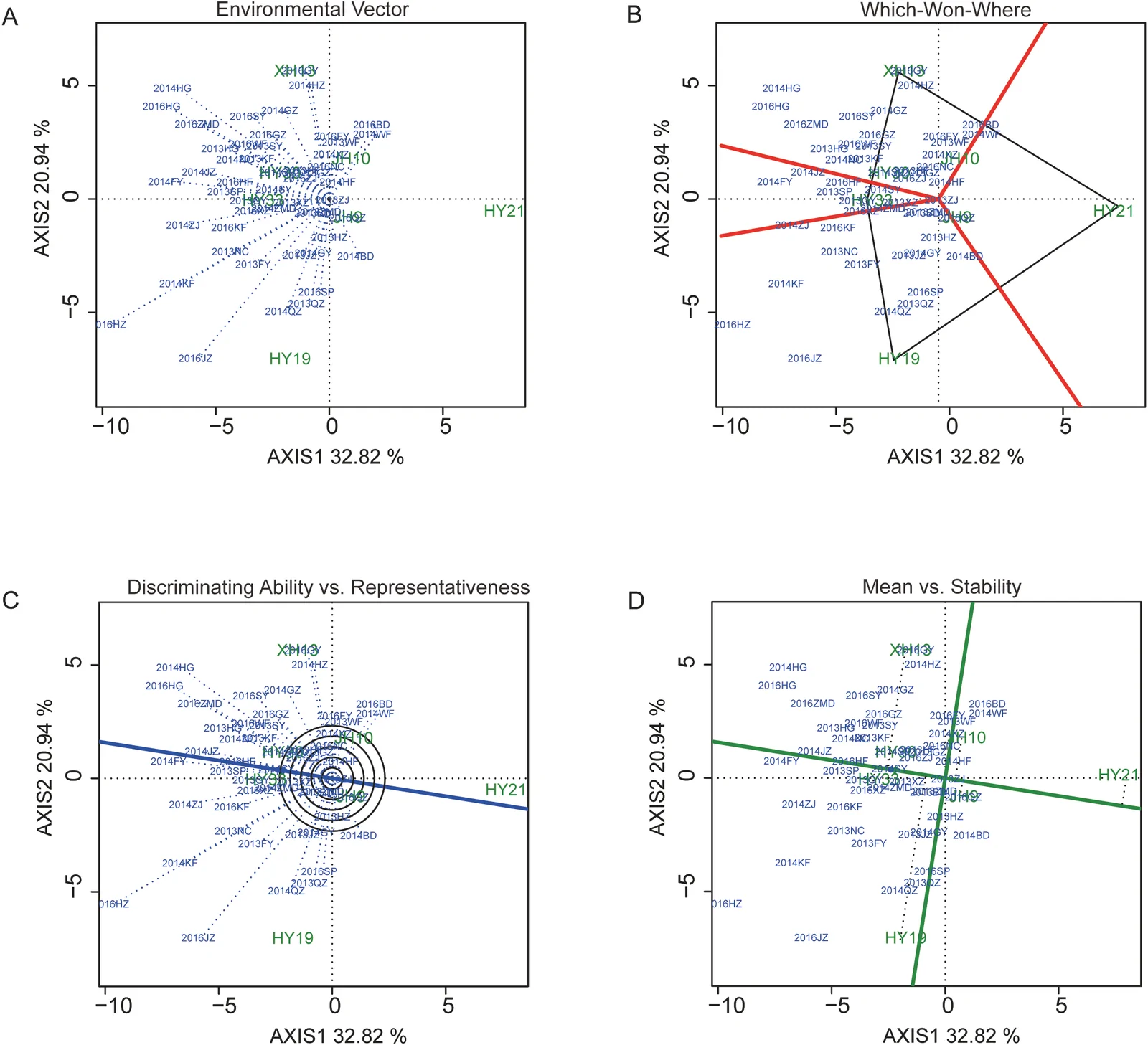
GGE biplot evaluations for oil content in 51 environments. Multi-environment GGE biplots of Environmental Vector **(A)**, Which-Won-Where **(B)**, Discriminating Ability vs. Representativeness and Mean vs. Stability **(D)** analysis for oil content trait.

For protein content, the first two axes explained 35.64% and 23.11% of total phenotypic variance, respectively. QZ, KF, and HG were strongly positively correlated across three years, whereas GY and BD were negatively correlated (**Figure 7A**). The “which‑won‑where” biplot divided the 51 environments into three mega‑environments (**Figure 7B**), with JH10, HY19, and HY21 as the optimal genotypes for mega‑environments containing 23, 18, and 10 environments, respectively (**Supplementary Table S2**).

**Figure 7:**
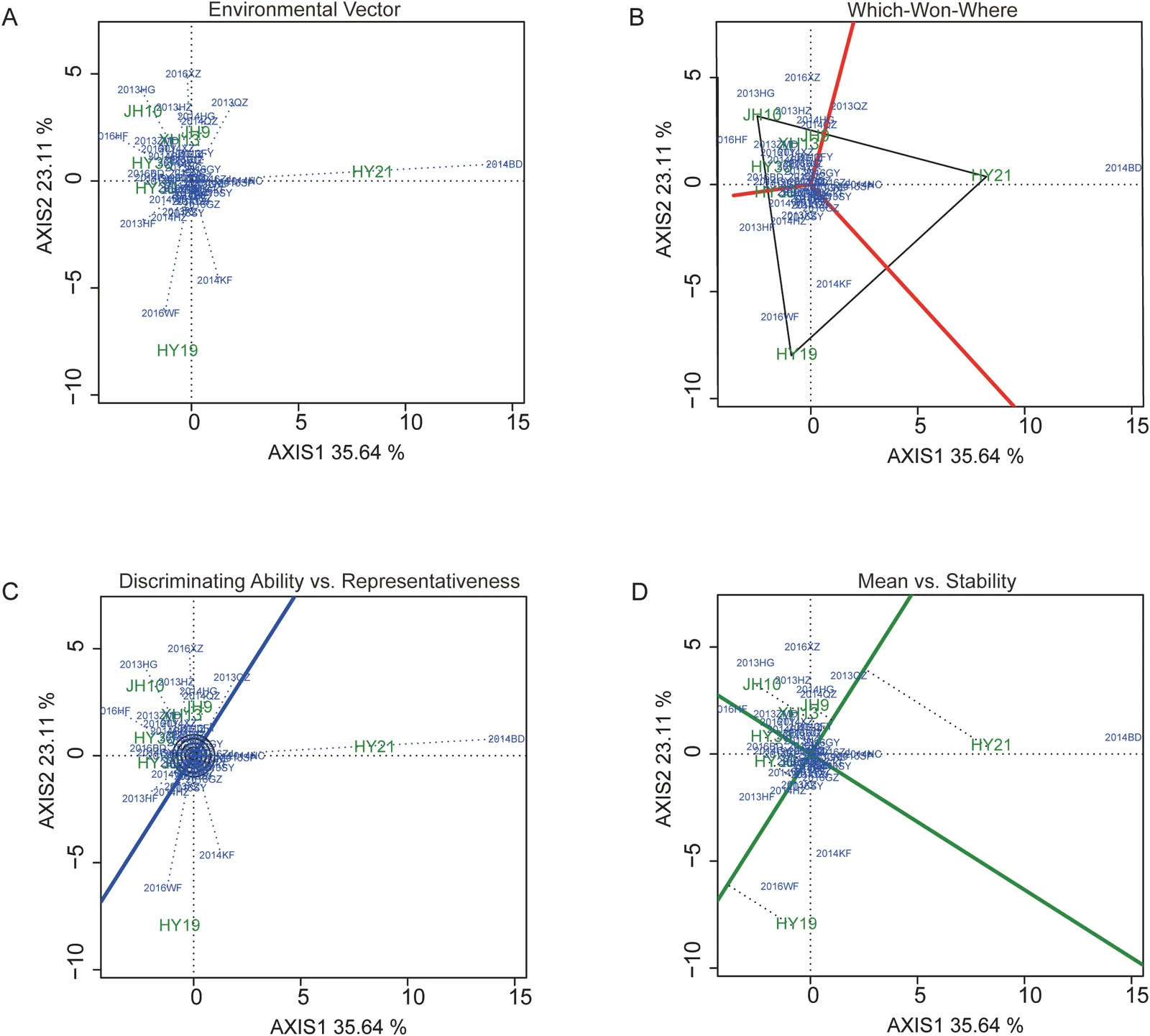
GGE biplot evaluations for protein content in 51 environments. Multi-environment GGE biplots of Environmental Vector **(A)**, Which-Won-Where **(B)**, Discriminating Ability vs. Representativeness and Mean vs. Stability **(D)** analysis for protein content trait.

Discrimination and representativeness analysis showed that 2013QZ had the highest correlation with the average environment axis, while 2014BD, 2016WF, and 2014KF had the strongest discriminatory power for protein content (**Figure 7C**). Mean‑stability analysis indicated that HY21 had the highest protein content but relatively low stability, whereas JH9 had the highest stability and the second‑highest protein content across 51 environments (**Figure 7D**).

For sucrose content, the first two axes accounted for 26.24% and 21.11% of total phenotypic variance, respectively. KF and FY were strongly positively correlated across three years, indicating stable climatic conditions and suitability for peanut cultivation (**Figure 8A**). The “which‑won‑where” biplot separated the 51 environments into four mega‑environments (**Figure 8B**), with HY21, HY33, HY19, and XH13 as the optimal genotypes for mega‑environments containing 30, 7, 7, and 7 environments, respectively (**Supplementary Table S2**). 2016HG and 2014HZ showed the strongest discriminatory power, while 2014HG and 2016GY were the most representative, with the highest correlation with the average environment axis (**Figure 8C**). Mean‑stability analysis demonstrated that HY21 had both the highest sucrose content and the strongest multi‑environmental stability (**Figure 8D**).

**Figure 8:**
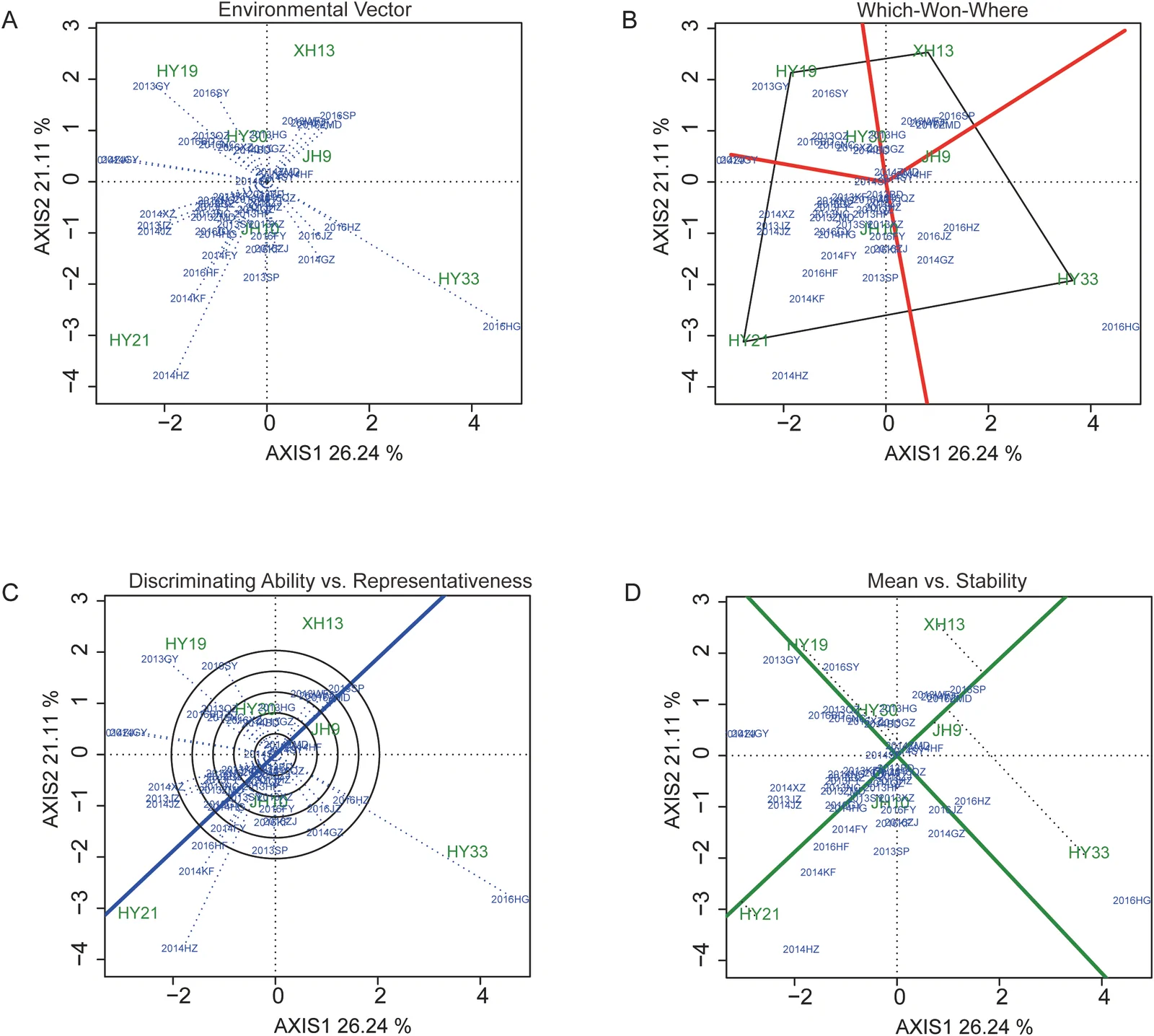
GGE biplot evaluations for sucrose content in 51 environments. Multi-environment GGE biplots of Environmental Vector **(A)**, Which-Won-Where **(B)**, Discriminating Ability vs. Representativeness and Mean vs. Stability **(D)** analysis for sucrose content trait.

## Discussion

Peanut is a widely cultivated cash and oil crop in China, and regional and meteorological factors, which serve as the most critical environmental drivers, exert a pivotal influence on peanut kernel quality traits. Its cultivation areas, which span temperate and subtropical monsoon climates, are geographically divided into six major agro-ecological zones. The Huang-Huai-Hai production area, which mainly includes Shandong, Henan, and Hebei, is characterized by a temperate monsoon climate with abundant sunshine duration and is mainly used for planting large-grain and high-oleic-acid varieties. Owing to the fertile soil of the Yellow River alluvial plains, peanut production in the Huang-Huai-Hai production area always exhibits excellent yield and quality (**Figures 1B**, **4**). The South China production area, which mainly includes Guangdong, Guangxi, Fujian, and Hainan, features a tropical and subtropical climate with high temperature, abundant rainfall, and a long frost-free period, which makes it suitable for producing processed peanuts such as salted, dried, and roasted peanuts. The Yangtze River Basin production area, which mainly includes Hubei, Hunan, and Jiangxi, has a subtropical monsoon climate with abundant rainfall and high humidity in summer, where summer-sown peanuts are dominant under wheat or rape stubble rotation systems. The Northeast Plain production area, which covers Liaoning and Jilin, features a temperate continental climate with cold winters and a short growing period, where spring-sown and early-maturing varieties dominate peanut production. The Yunnan-Guizhou Plateau production area, which belongs to a plateau mountain climate with significant vertical differences and large diurnal temperature variations, is where peanuts are planted in hills and mountains at an altitude of 1,000–2,000 m with spring sowing as the main practice. The Northwestern production area, which includes Xinjiang and Gansu, is under a temperate continental arid climate with strong sunshine, limited precipitation, and large diurnal temperature variation.

Complex meteorological and geographical factors act jointly to affect peanut yield and quality traits in China, and meteorological factors such as light, temperature, and water, which are key environmental regulators, contribute to divergent peanut growth performances (Ai et al., 2025; Jongrungklang et al., 2012; Kumar et al., 2012; Qi et al., 2025; Zhen et al., 2024). Meanwhile, climate change, which has become a major threat, affects the stable production of peanuts ( (Khan et al., 2009; Wei et al., 2022; Wheeler and von Braun, 2013; Ye et al., 2013). Therefore, to clarify the effects of meteorological and geographical factors on peanut kernel quality traits, we performed a multi-environment evaluation using phenotypic data of seven major popularized varieties planted at 17 locations over three years, which generated a total of 51 environments, together with synchronized meteorological and geographical data for each environment. The results showed that oil content was extremely significantly and positively correlated with longitude, accumulated temperature ≥ 10 °C, and total sunshine duration across the entire growth period. Protein content was extremely significantly and positively correlated with accumulated temperature ≥ 10 °C and mean daily temperature across the entire growth period, and significantly and positively correlated with longitude and mean daily temperature difference. Sucrose content was extremely significantly and negatively correlated with longitude, latitude, accumulated temperature ≥ 10 °C, and total sunshine duration across the entire growth period. Multi-environment phenotypic data revealed that oil and protein contents displayed a spatial trend of being higher in eastern China and lower in western China, while sucrose content showed a trend of being higher in southeastern China and lower in northwestern China. Furthermore, correlation results between quality traits and meteorological factors suggested the feasibility of simultaneous improvement in peanut oil and protein contents. Two-way ANOVA showed that environmental factors accounted for approximately 60% of the total variation in peanut oil, protein, and sucrose contents, which indicates that most differences in peanut kernel quality traits across multiple environments are caused by environmental factors. For peanut oil, protein, and sucrose contents, environmental effects are comparable to genetic effects. Single meteorological factor performed low linear regression and non-significant with the three kernel quality traits those indicated that a single factor cannot adequately explain the variation in quality traits, and multi-factor interactions are a key focus for future research.

Genotype, environment, and genotype-by-environment interaction (GEI) have long been research focuses for crop breeders, and GEI, which represents one of the most complex constraints, challenges breeding programs aiming to identify high-quality and stable peanut genotypes. Recently, GEI and GGE biplot analyses have been applied in studies of peanut yield, kernel quality traits, biotic stress tolerance, phosphorus-efficient genotype identification, and sensory and biochemical composition (Tang et al., 2025; Wang et al., 2024; Zhen et al., 2024, Zhen et al., 2023). In the US, Wang et al. (2024) used 52 peanut germplasm accessions, found that Florida produced the highest oil content and the lowest protein content with a negative correlation between oil and protein contents. In China, Tang et al. (2025), who used 11 germplasm accessions and two varieties planted across three environments located in the Huang-Huai-Hai production area (Weifang) and the Northeast Plain production area (Jinzhou and Fuxin), found that Weifang showed the best oil and protein contents while Jinzhou had the highest sucrose content. Compared with their findings, our results are consistent in that protein and oil contents are positively correlated with accumulated temperature, whereas sucrose content is negatively correlated with accumulated temperature.

In this study, we used seven popularized peanut varieties planted at 17 locations distributed across five major peanut production areas. For oil content, the 51 environments were classified into four mega-environments, in which XH13, HY33, HY19, and HY21 were the optimal genotypes, which indicates that these varieties may perform better in corresponding environments or locations. Meanwhile, HY33 and HY30 showed the highest oil content and strongest multi-environmental stability. For protein content, the 51 environments were divided into three mega-environments, in which HY19, HY21, and JH10 were the optimal genotypes with the best performance in each mega-environment. HY21 exhibited the highest mean protein content but relatively low multi-environmental stability, whereas JH9 ranked second in protein content with far superior multi-environmental stability. For sucrose content, the GGE biplot partitioned the environments into four sectors, in which HY19, HY21, HY33, and XH13 were the optimal genotypes with the best sucrose performance in each mega-environment. HY21 was the optimal genotype in 30 environments across 5 production areas, meanwhile, it showed the highest mean sucrose content and strongest multi-environmental stability. Based on a comprehensive analysis of the three peanut kernel quality traits, HY33 was identified as the superior stable oil variety and HY21 was the top performer for protein and sucrose, which suggests that they are suitable for wide promotion across multiple production areas and can be used as elite breeding parents for developing trait-specific varieties.

Meteorological and geographical factors, which are critical indicators, play essential roles in modern agricultural cultivation planning. Ecological regions for cotton fiber quality have been delineated in China’s major cotton planting regions and in Spain, and ecological zoning has also been established for barley, maize, and other crops (Baxevanos et al., 2008). In the modern peanut industry, temperature has been used as a zoning indicator, and climatic zones suitable for different ecological peanut varieties in China have been identified. In addition, a national peanut suitability region map has been developed based on the interactive effects of climatic and soil factors on peanut cultivation. Therefore, analyzing the regional characteristics of peanut growth and ecological resources, which is crucial for evaluating peanut ecological suitability, supports rational planting division, peanut cropping structure adjustment, and optimized production layout tailored to local conditions. Among the three-year evaluation, GZ, KF, HG, and QZ showed environmental stability for oil content; QZ, KF, and HG showed environmental stability for protein content; and KF and FY exhibited environmental stability for sucrose content. By integrating the three quality traits, we found that KF had relatively stable climatic conditions, and peanut kernel quality traits in KF showed good repeatability and strong representativeness across diverse locations. These findings provide supportive information and evidence for peanut industry planning in China.

## Conclusion

In this study, a multi-environment evaluation was conducted, which investigated peanut kernel quality traits of seven popularized peanut varieties at 17 locations over three years. The cultivation trials, which covered five major peanut-producing areas and nearly all major peanut-producing provinces in China, spanned a geographical range from 21.09°N to 43.07°N and from 106.08°E to 124.23°E. The evaluation demonstrated that entire growth period accumulated temperature ≥ 10 °C and total sunshine duration can enhance oil content, while accumulated temperature ≥ 10 °C, mean daily temperature, and mean daily temperature difference can increase protein content. In contrast, sucrose content was extremely significantly and negatively correlated with accumulated temperature ≥ 10 °C and total sunshine duration. Oil and protein contents showed a spatial pattern of being higher in eastern China and lower in western China, which revealed the mechanism that Yangtze River Basin and Huang-Huai-Hai production areas had the highest oil and protein contents. While sucrose content displayed a pattern of being higher in southeastern China and lower in northwestern China. ANOVA revealed that environmental factors contributed approximately 60% of the total variation in peanut oil, protein, and sucrose contents, which suggests that environmental effects are comparable to genetic effects on peanut kernel quality traits. GGE stability analysis confirmed HY33 as the optimal stable cultivar for high oil concentration, while HY21 combined superior protein levels with maximum sucrose content and cross-region stability. In addition, KF had relatively stable climatic conditions and the strongest representativeness across diverse locations. These findings supply empirical guidance for peanut breeding and regional industrial layout optimization both domestically and internationally.

## Data Availability

The original contributions presented in the study are included in the article/**Supplementary Material**. Further inquiries can be directed to the corresponding author.

## Supplementary material

Supplementary Figure 1 Linear regression between environmental factors and quality traits. Linear regression for the eight environmental factors and oil **(A)**, protein **(B)** and sucrose **(C)** content traits.

Supplementary Table 1 Annual statistics of phenotypic data of peanut varieties.

Supplementary Table 2 The number of mega-environments, environment and the genotypes by the Which-Won-Where View.

## References

[B1] Ai X. Wang S. Dai R. Ma X. Li C. Zhong C. . (2025). Dynamic changes of seed development, oil accumulation and fatty acid composition in peanut under soil water deficit. Plant Physiol. Biochem. 219, 109336. doi: 10.2139/ssrn.4989447 39616800

[B2] Ansarifard I. Mostafavi K. Khosroshahli M. Reza Bihamta M. Ramshini H. (2020). A study on genotype-environment interaction based on GGE biplot graphical method in sunflower genotypes (Helianthus annuus L.). Food Sci. Nutr. 8, 3327–3334. doi: 10.1002/fsn3.1610 32724597 PMC7382153

[B3] Bai J. Yan W. Wang Y. Yin Q. Liu J. Wight C. . (2018). Screening oat genotypes for tolerance to salinity and alkalinity. Front. Plant Sci. 9, 1302. doi: 10.3389/fpls.2018.01302 30333838 PMC6176118

[B4] Baxevanos D. Goulas C. Rossi J. Braojos E. (2008). Separation of cotton cultivar testing sites based on representativeness and discriminating ability using GGE biplots. Agron. J. 100, 1230–1236. doi: 10.2134/agronj2007.0363

[B5] Chen X. Wu W. Zhang W. (2019). Application of grey correlation theory and regression analysis in influence of meteorological factors on spring peanut yield:Taking Guzhen country of Anhui province as an example. Agric. Eng 9, 111–115.

[B6] Chintalapati P. Balakrishnan D. Venu Gopal Nammi T. V. Javvaji S. Muthusamy S. K. Lella Venkata S. R. . (2019). Phenotyping and genotype × environment interaction of resistance to leaffolder, Cnaphalocrocis medinalis Guenee (Lepidoptera: Pyralidae) in rice. Front. Plant Sci. 10, 49. doi: 10.3389/fpls.2019.00049 30833948 PMC6387916

[B7] David Kulyakwave P. Xu S. Wen Y. (2020). Impact of meteorological factors on rice growth stages and yield. Pertanika J. Sci. Technol. 28, 1009–1026.

[B8] Dwivedi S. L. Nigam S. N. Rao R. C. N. (2000). Photoperiod effects on seed quality traits in peanut. Crop Sci. 40, 1223–1227. doi: 10.2135/cropsci2000.4051223x

[B9] Han Y. Lu H. Qiao D. (2023). Integrated effects of meteorological factors, edaphic moisture, evapotranspiration, and leaf area index on the net primary productivity of winter wheat ‑ summer maize rotation system. Field Crops Res. 302, 109080. doi: 10.1016/j.fcr.2023.10908042574925

[B10] Hristov N. Mladenov N. Djuric V. Kondic-Spika A. Marjanovic-Jeromela A. Simic D. (2010). Genotype by environment interactions in wheat quality breeding programs in southeast Europe. Euphytica 174, 315–324. doi: 10.1007/s10681-009-0100-830311153

[B11] Huang Q. Wang L. Chen Z. Liu H. (2016). Effects of meteorological factors on different grades of winter wheat growth in the Huang-Huai-Hai Plain, China. J. Integr. Agric. 15, 2647–2657. doi: 10.1016/s2095-3119(16)61464-8

[B12] Jat R. K. Singh P. Jat M. L. Dia M. Sidhu H. S. Jat S. L. . (2018). Heat stress and yield stability of wheat genotypes under different sowing dates across agro-ecosystems in India. Field Crops Res. 218, 33–50. doi: 10.1016/j.fcr.2017.12.02042574925

[B13] Jiayu Z. Shiwei X. Ganqiong L. Yongen Z. Jianzhai W. Jiajia L. (2018). The influence of meteorological factors on wheat and rice yields in China. Crop Sci. 58, 837–852. doi: 10.2135/cropsci2017.01.0048er

[B14] Jongrungklang N. Toomsan B. Vorasoot N. Jogloy S. Boote K. J. Hoogenboom G. . (2012). Classification of root distribution patterns and their contributions to yield in peanut genotypes under mid-season drought stress. Field Crops Res. 127, 181–190. doi: 10.1016/j.fcr.2011.11.02342574925

[B15] Katuuramu D. N. Luyima G. B. Nkalubo S. T. Wiesinger J. A. Kelly J. D. Cichy K. A. (2020). On-farm multi-location evaluation of genotype by environment interactions for seed yield and cooking time in common bean. Sci. Rep. 10, 3628. doi: 10.1038/s41598-020-60087-2 32107403 PMC7046726

[B16] Kebede B. A. Getahun A. (2017). Adaptability and stability analysis of groundnut genotypes using AMMI model and GGE-biplot. J. Crop Sci. Biotechnol. 20, 343–349. doi: 10.1007/s12892-017-0061-030311153

[B17] Khan S. Hanjra M. A. Mu J. (2009). Water management and crop production for food security in China: A review. Agric. Water Manage. 96, 349–360. doi: 10.1016/j.agwat.2008.09.02242574925

[B18] Kumar U. Singh P. Boote K. J. (2012). “ Chapter two - Effect of climate change factors on processes of crop growth and development and yield of groundnut (Arachis hypogaea L.),” in Advances in Agronomy, vol. 116 . Ed. Sparks D. L. (San Diego, CA: Academic Press), 41–69.

[B19] Lamichaney A. Parihar A. K. Hazra K. K. Dixit G. P. Katiyar P. K. Singh D. . (2021). Untangling the influence of heat stress on crop phenology, seed set, seed weight, and germination in field pea (Pisum sativum L.). Front. Plant Sci. 12, 635868. doi: 10.3389/fpls.2021.635868 33854520 PMC8040956

[B20] Lingaiah N. Sudharshanam A. Thirumala Rao V. Prashanth Y. Kumar M. Reddy P. . (2020). AMMI biplot analysis in cotton (Gossypium hirsutum L.) genotypes for genotype x environment interaction at four agro-ecologies in Telangana State. Curr. J. Appl. Sci. Technol. 39 (15), 98–103. doi: 10.9734/cjast/2020/v39i153072242687847

[B21] Nagesh Kumar M. V. Ramya V. Govindaraj M. Sameer Kumar C. V. Maheshwaramma S. Gokenpally S. . (2021). Harnessing sorghum landraces to breed high-yielding, grain mold-tolerant cultivars with high protein for drought-prone environments. Front. Plant Sci. 12, 659874. doi: 10.3389/fpls.2021.659874 34276722 PMC8279770

[B22] Oteng-Frimpong R. Dakora F. D. (2019). Multienvironment testing for trait stability and G × E interaction on N2 fixation, plant development, and water-use efficiency of 21 elite groundnut (Arachis hypogaea L.) genotypes in the Guinea Savanna. Front. Plant Sci. 10, 2019. doi: 10.3389/fpls.2019.01070 31572406 PMC6751404

[B23] Qi S. Li H. Bi Y. Zou X. Zhang X. Yu X. . (2025). Moderate source-reduction improves peanut yield and quality through modulating photosynthesis, source-sink equilibrium, and secondary metabolisms. Plant Physiol. Biochem. 229, 110415. doi: 10.1016/j.plaphy.2025.110415 40850301

[B24] Serneels S. Verdonck T. (2008). Principal component analysis for data containing outliers and missing elements. Comput. Stat. Data Anal. 52, 1712–1727. doi: 10.1016/j.csda.2007.05.02442574925

[B25] Setimela P. S. Vivek B. Bänziger M. Crossa J. Maideni F. (2007). Evaluation of early to medium maturing open pollinated maize varieties in SADC region using GGE biplot based on the SREG model. Field Crops Res. 103, 161–169. doi: 10.1016/j.fcr.2007.05.01042574925

[B26] Shukla S. Mishra B. K. Mishra R. Siddiqui A. Pandey R. Rastogi A. (2015). Comparative study for stability and adaptability through different models in developed high thebaine lines of opium poppy (Papaver somniferum L.). Ind. Crops Prod. 74, 875–886. doi: 10.1016/j.indcrop.2015.05.07642574925

[B27] Tang Y. Cao S. Zhang C. Hu W. Zhou H. Wang M. . (2025). Genotype-by-environment interaction influencing sensory attributes and biochemical components of peanuts from China. Front. Plant Sci. 16, 1609969. doi: 10.3389/fpls.2025.1609969 40822717 PMC12354597

[B28] Vida G. Cséplő M. Rakszegi M. Bányai J. (2022). Effect of multi-year environmental and meteorological factors on the quality traits of winter durum wheat. Plants (Basel) 11 (1), 113. doi: 10.3390/plants11010113 35009116 PMC8747632

[B29] Vlachostergios D. N. Roupakias D. G. (2008). Response to conventional and organic environment of thirty-six lentil (Lens culinaris Medik.) varieties. Euphytica 163, 449–457. doi: 10.1007/s10681-008-9712-730311153

[B30] Wang Y. Ahmed A. Xu S. Yu W. Liu J. (2013). Meteorological impact on peanut yield in China. BTAIJ 8, 1251–1257. doi: 10.3923/jas.2013.2740.2743

[B31] Wang J. Liu W. Yin D. (2025). Impacts of integrated meteorological and agricultural drought on global maize yields. Agric. Water Manage. 318, 109727. doi: 10.1016/j.agwat.2025.10972742574925

[B32] Wang M. L. Tonnis B. Li X. Benke R. Huang E. Tallury S. . (2024). Genotype, environment, and their interaction effects on peanut seed protein, oil, and fatty acid content variability. Agron. J. 116, 1440–1454. doi: 10.1002/agj2.2155942587375

[B33] Wang J. Zhang X. Su L. Li H. Zhang L. Wei J. (2020). Global warming effects on climate zones for wine grape in Ningxia region, China. Theor. Appl. Climatol. 140, 1527–1536. doi: 10.1007/s00704-020-03170-y30311153

[B34] Wei S. Li K. Yang Y. Wang C. Liu C. Zhang J. (2022). Comprehensive climatic suitability evaluation of peanut in Huang-Huai-Hai region under the background of climate change. Sci. Rep. 12, 11350. doi: 10.1038/s41598-022-15465-3 35790844 PMC9256610

[B35] Wheeler T. von Braun J. (2013). Climate change impacts on global food security. Science 341, 508–513. doi: 10.1126/science.1239402 23908229

[B36] Yan W. Hunt L. A. (2001). Interpretation of genotype × environment interaction for winter wheat yield in Ontario. Crop Sci. 41, 19–25. doi: 10.2135/cropsci2001.41119x

[B37] Yan W. Rajcan I. (2002). Biplot analysis of test sites and trait relations of soybean in Ontario. Crop Sci. 42, 11–20. doi: 10.2135/cropsci2002.1100 11756248

[B38] Ye L. Xiong W. Li Z. Yang P. Wu W. Yang G. . (2013). Climate change impact on China food security in 2050. Agron. Sustain. Dev. 33, 363–374. doi: 10.1007/s13593-012-0102-030311153

[B39] Zhang Y. Wang P. Li Y. Han D. Wu D. Liu S. . (2025). Characterization of meteorological and agricultural drought for summer maize and its climatic drivers. Atmos Res. 326, 108329. doi: 10.1016/j.atmosres.2025.10832942574925

[B40] Zhao J. Li K. Wang R. Tong Z. Zhang J. (2019). Yield data provide new insight into the dynamic evaluation of maize’s climate suitability: A case study in Jilin Province, China. Atmosphere 10, 305. doi: 10.3390/atmos1006030530654563

[B41] Zhen X. Huo W. Sanz-Saez A. Miao Y. Chen C. Y. Batchelor W. D. (2024). Drought-tolerant peanut (Arachis hypogaea L.) varieties can mitigate negative impacts of climate change on yield in the Southeastern U.S. Comput. Electron. Agric. 224, 109105. doi: 10.1016/j.compag.2024.10910542574925

[B42] Zhen X. Huo W. Tian D. Zhang Q. Sanz-Saez A. Chen C. Y. . (2023). County level calibration strategy to evaluate peanut irrigation water use under different climate change scenarios. Eur. J. Agron. 143, 126693. doi: 10.1016/j.eja.2022.12669342574925

